# *Cleome rutidosperma* and *Euphorbia thymifolia* Suppress Inflammatory Response via Upregulation of Phase II Enzymes and Modulation of NF-κB and JNK Activation in LPS-Stimulated BV2 Microglia

**DOI:** 10.3390/ijms17091420

**Published:** 2016-08-27

**Authors:** Hsiou-Yu Ding, Pei-Shan Wu, Ming-Jiuan Wu

**Affiliations:** 1 Department of Cosmetic Science, Chia Nan University of Pharmacy and Science, Tainan 717, Taiwan; hsiou221@yahoo.com.tw; 2 Department of Biotechnology, Chia Nan University of Pharmacy and Science, Tainan 717, Taiwan; dc7575@gmail.com

**Keywords:** *Cleome rutidosperma*, *Euphorbia thymifolia*, microglial cells, HO-1, JNK, NF-κB

## Abstract

*Cleome rutidosperma* DC. and *Euphorbia thymifolia* L. are herbal medicines used in traditional Indian and Chinese medicine to treat various illnesses. Reports document that they have antioxidant and anti-inflammatory activities; nonetheless, the molecular mechanisms involved in their anti-inflammatory actions have not yet been elucidated. The anti-neuroinflammatory activities and underlying mechanisms of ethanol extracts of *Cleome rutidosperma* (CR) and *Euphorbia thymifolia* (ET) were studied using lipopolysaccharide (LPS)-stimulated microglial cell line BV2. The morphology changes and production of pro-inflammatory mediators were assayed. Gene expression of inflammatory genes such as inducible nitric oxide synthase (iNOS), cyclooxygenase (COX)-2, interleukin (IL)-1β, and CC chemokine ligand (CCL)-2, as well as phase II enzymes such as heme oxygenase (HO)-1, the modifier subunit of glutamate cysteine ligase (GCLM) and NAD(P)H quinone dehydrogenase 1 (NQO1), were further investigated using reverse transcription quantitative-PCR (RT-Q-PCR) and Western blotting. The effects of CR and ET on mitogen activated protein kinases (MAPKs) and nuclear factor (NF)-κB signaling pathways were examined using Western blotting and specific inhibitors. CR and ET suppressed BV2 activation, down-regulated iNOS and COX-2 expression and inhibited nitric oxide (NO) overproduction without affecting cell viability. They reduced LPS-mediated tumor necrosis factor (TNF) and IL-6 production, attenuated IL-1β and CCL2 expression, but upregulated HO-1, GCLM and NQO1 expression. They also inhibited p65 NF-κB phosphorylation and modulated Jun-N terminal kinase (JNK) activation in BV2 cells. SP600125, the JNK inhibitor, significantly augmented the anti-IL-6 activity of ET. NF-κB inhibitor, Bay 11-7082, enhanced the anti-IL-6 effects of both CR and ET. Znpp, a competitive inhibitor of HO-1, attenuated the anti-NO effects of CR and ET. Our results show that CR and ET exhibit anti-neuroinflammatory activities by inhibiting pro-inflammatory mediator expression and production, upregulating HO-1, GCLM and NQO1, blocking NF-κB and modulating JNK signaling pathways. They may offer therapeutic potential for suppressing overactivated microglia and alleviating neurodegeneration.

## 1. Introduction

Inflammation is a complex response that presents as series of vascular and cellular reactions triggered by injury or infection. Resident macrophages belong to the innate immune system responsible for the first line of defense against injury and infection. Microglia, the resident macrophage cells in the central nervous system (CNS), effectively phagocyte plaques, damaged or unnecessary neurons and synapses, and infectious agents to maintain neuronal homeostasis [1,2]. In response to pathophysiological brain insults, microglia become activated as characterized by shape changes and production of immune modulators in order to regulate tissue repair and recovery [3]. Sustained activation of microglial cells cause overproduction of various pro-inflammatory cytokines and mediators, which could lead to neurodegenerative diseases such as Alzheimer’s disease (AD), Huntington’s disease (HD), Parkinson’s disease (PD), and amyotrophic lateral sclerosis (ALS) [1].

NO (nitric oxide) is a very potent activator of Keap1-Nrf2 (NF-E2-related factor 2), which induces the expression of Phase II detoxification enzymes to adapt to oxidative stress conditions [4]. The Phase II enzymes include heme oxygenase (HO)-1, glutamate cysteine ligase (GCL), NAD(P)H quinone dehydrogenase 1 (NQO1) and other antioxidant enzymes [5]. The HOs are the rate-limiting enzymes in the degradation of excess heme and generation of biliverdin, ferrous ion, and carbon monoxide (CO) [6]. HO-1 is normally expressed at low levels but is induced in response to a variety of stimuli to protect cells [7]. A substantial body of evidence demonstrates that the induction of HO-1 is a vital step in the cellular adaptation to pathological stress [8]. Many inducers of HO-1 have been reported to have anti-inflammatory properties [8,9,10,11,12], possibly due to the antioxidant functions of enzymatic products.

Mitogen-activated protein kinases (MAPK) are protein kinases involved in relaying a diverse array of stimuli from the cell membrane to the nucleus by means of a cascade of phosphorylation events [13]. The extracellular signal-regulated kinases (ERKs) are widely expressed and involved in the regulation of proliferation, differentiation and cell cycle progression [14]. Phospho-c-Jun N-terminal kinases (JNKs) are activated primarily by cytokines and exposure to environmental stress [15]. The p38 MAPK is activated in immune cells by inflammatory cytokines and is critical for normal immune and inflammatory response [14].

The nuclear factor (NF)-κB is a critical component in regulation of inflammatory responses. NF-κB can form different dimers, but a p50/p65 heterodimer is the most common one [16]. The central mechanism for NF-κB activation is phosphorylation of inhibitors of NF-κB (IκBs) by IκB kinase (IκKs) and leads to the proteasomal degradation of IκBs and allowing NF-κB to enter the nucleus. Besides the phosphorylation and degradation of IκBs, post-translational phosphorylation of NF-κB is also important for its activity [17]. Special attention has been paid to phosphorylation of p65 at Ser^536^, which is involved in regulation of transcriptional activity, nuclear import and protein stability [18].

There is a growing interest in searching for natural products with anti-inflammatory activities [19]. *Cleome rutidosperma* DC. is an herb native to West Africa that has spread and naturalized in various parts of the world, and is used in Indian medicine to treat paralysis, epilepsy, convulsions, spasm, pain and skin disease [20], with its antiplasmodial, antimicrobial, diuretic, antioxidant, analgesic, anti-pyretic, anti-arthritic and wound healing activities having been reported in the literature [20,21,22,23,24,25]. *Euphorbia thymifolia* L. has many synonyms such as *Anisophyllum thymifolium* (L.) Haw., *Aplarina microphylla* (Lam.) Raf., *Chamaesyce microphylla* (Lam.) Soják, *C. rubrosperma* (Lotsy) Millsp., *C. thymifolia* f. *suffrutescens* (Boiss.) Hurus., *E. afzelii* N.E.Br., *E. botryoides* Noronha, *E. rubicunda* Blume, *E. rubrosperma* Lotsy, *E. thymifolia* f. *laxifoliata* Chodat and Hassl., and so on [26]. It is a heat-clearing remedy in the Chinese medicine and commonly used for the treatment of acute enteritis, diarrhea, atopic dermatitis and inflammatory diseases. Previous studies reveal that it has various kinds of bioactivities, including antioxidant [27,28], anti-viral [27,29,30], anti-microbial [31], and anti-earthworm [32]. However, the molecular pharmacological mechanism underlying their anti-inflammatory activities has never been reported.

Immortalized microglia BV2 stimulated with lipopolysaccharide (LPS) is a suitable model for evaluating neuroinflammatory responses [33,34,35,36]. Because *C. rutidosperma* and *E. thymifolia* share similar bioactivities and are used in treating fever or inflammatory diseases in folk medicine, the aim of this study is to investigate their anti-neuroinflammatory effects and the underlying molecular mechanism in BV2 cells.

## 2. Results

### 2.1. Ethanol Extracts of C. rutidosperma (CR) and E. thymifolia (ET) Inhibited Nitric Oxide (NO) Production and Activation in Lipopolysaccharide (LPS)-Treated BV2 Cells

**Figure 1:**
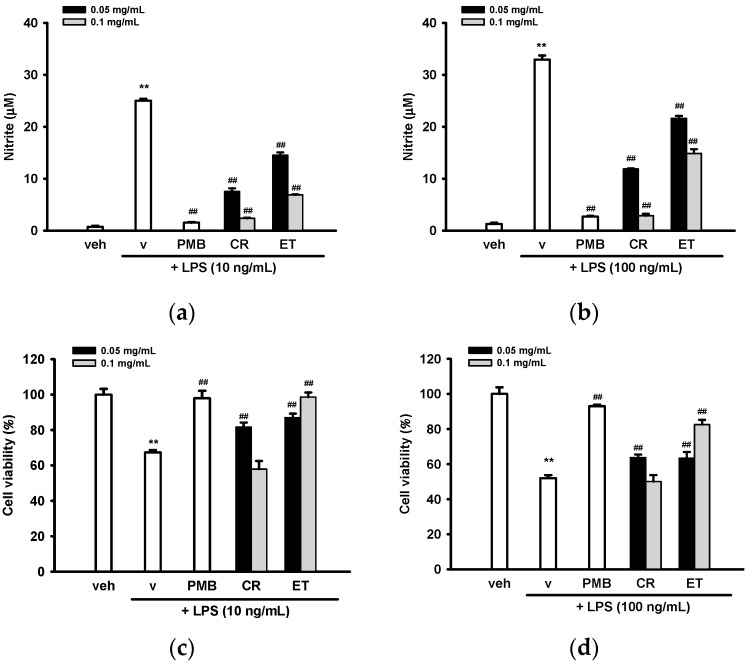

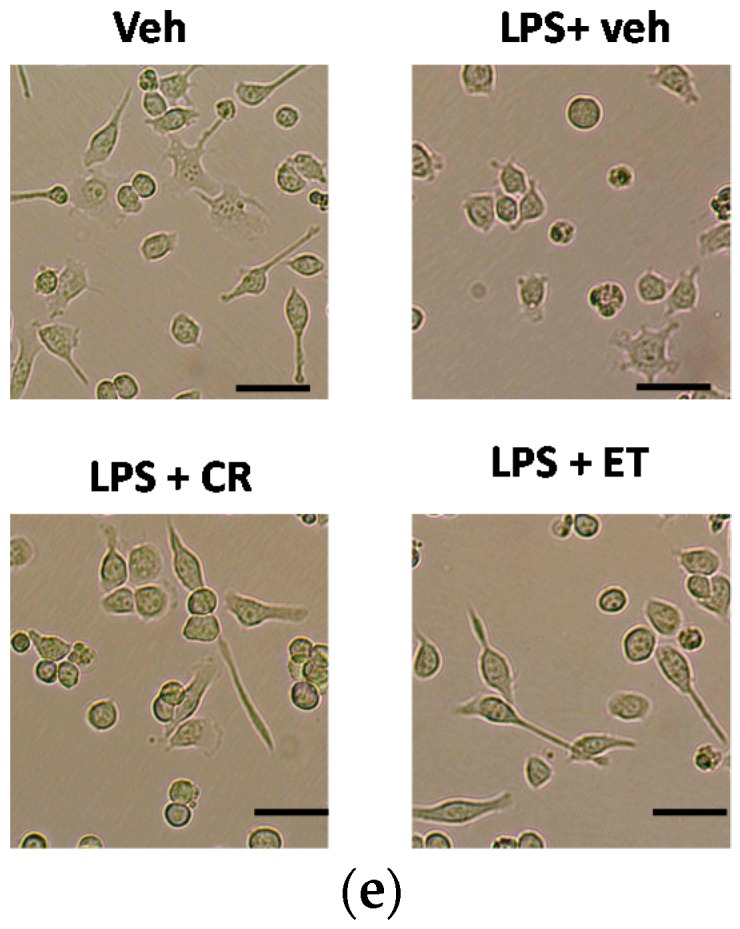
Effects of ethanol extracts of *C. rutidosperma* (CR) and *E. thymifolia* (ET) on lipopolysaccharide (LPS)-stimulated nitric oxide (NO) production and activation. BV2 cells were pre-treated for 0.5 h with polymyxin B (PMB, 10 µg/mL), vehicle (0.1% ethanol), or the indicated concentration of extract, and then stimulated with LPS (10 or 100 ng/mL) for 20 h. (**a**,**b**) The nitrite production in supernatant was determined by the Griess reagent; (**c**,**d**) The cell viability was analyzed by MTT assay. Data are represented as the mean ± SD (*n* = 3). Statistical differences are presented ** *p* < 0.01 compared with the vehicle control (without LPS) and ## *p* < 0.01 compared with the LPS-treated vehicle; (**e**) Representative images of BV2 microglia incubated for 20 h with vehicle (0.1% ethanol), LPS (10 ng/mL) plus vehicle (0.1% ethanol), LPS (10 ng/mL) + CR (0.05 mg/mL) or LPS (10 ng/mL) + ET (0.05 mg/mL). Images were acquired with Nikon Eclipse Ti-E inverted microscope (Scale bar, 50 µm).

To test whether CR and ET can function as inhibitors for NO release, BV2 cells were pre-treated with vehicle (0.1% ethanol), CR, or ET for 30 min followed by LPS (10 or 100 ng/mL) insult for a further 20 h. Polymyxin B (PMB, 10 µg/mL), a cyclic cationic polypeptide antibiotic, which binds to lipid A, served as a control LPS inhibitor. To set the optimal concentrations of CR and ET, we started with various concentrations of CR and ET ranging from 0.025–0.2 mg/mL with 1:2 serial dilutions. Our preliminary data showed that CR and ET at 0.025 mg/mL did not exert anti-NO activity significantly, while at 0.2 mg/mL caused significant cell death. As a result, 0.05 and 0.1 mg/mL of CR and ET were chosen for the experiments. Figure 1a,b shows that 10 and 100 ng/mL LPS plus vehicle stimulated NO production from basal levels (0.7–1.3 µM) to 25.0 ± 0.4 and 33.0 ± 0.8 µM, respectively. CR and ET (0.05–0.1 mg/mL) dose-dependently decreased 10 ng/mL LPS-induced NO production by 72%–93% and 43%–75%, respectively (*p* < 0.01). Slightly weaker inhibition (67%–93% for CR and 36%–57% for ET) was noted against 100 ng/mL LPS (*p* < 0.01). In comparison, PMB (10 µg/mL) almost completely inhibited 10 and 100 ng/mL LPS-mediated NO production.

It is well-known that LPS causes apoptosis in microglia [3], and 10 and 100 ng/mL LPS plus vehicle caused 32% and 48% BV2 cell death, respectively, as compared with the vehicle control (Figure 1c,d). Cell viabilities after 20 h of CR (0.1 mg/mL) co-treatment were more than 95% of those seen with the LPS plus vehicle groups, implying that the reduced NO caused by CR (0.1 mg/mL) treatment was not due to cytotoxicity. In comparison, CR (0.05 mg/mL), ET (0.05 and 0.1 mg/mL) and PMB (10 µg/mL) all significantly reversed LPS-induced cell death, indicating the cytoprotective effects may be attributable to their NO inhibitory activities. Because CR and ET in BV2 cells co-treated with 10 and 100 ng/mL LPS showed similar trend, only 10 ng/mL LPS was employed for the rest of experiments.

To assess whether the decreases in NO production in CR- and ET-treated microglia correlate with detectable functional properties, characterization of cell morphological changes was performed. In general, the basal microglia have ramified shapes at the resting state. Once activated by LPS, the cells quickly convert to round or amoeboid shapes [37]. Figure 1e shows that BV2 cells treated with vehicle had the typical branching shape with smaller soma. BV2 cells treated with LPS (10 ng/mL) plus vehicle for 20 h showed the enlargement of the microglial cell body with retracted filopodia. In comparison, co-incubation of LPS-treated cells with CR or ET (0.05 mg/mL) reversed the morphological changes caused by LPS and sustained the ramified shape with some filopodia.

### 2.2. Ethanol Extracts of C. rutidosperma and E. thymifolia Down-Regulated Protein Expression of Inducible Nitric Oxide Synthase (iNOS) and Cyclooxygenase (COX)-2

**Figure 2:**
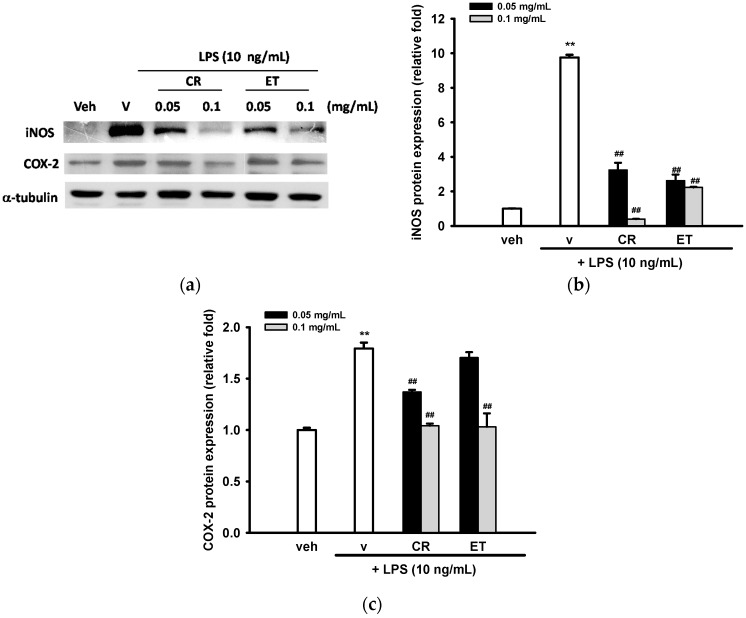
Effects of ethanol extracts of *C. rutidosperma* and *E. thymifolia* on iNOS and COX-2 expression in LPS-induced BV2 cells. (**a**) BV2 cells were cultured with indicated reagent in 6-well plates for 16 h and Western blotting of iNOS, COX-2 and α-tubulin were performed, as described in Materials and Methods. Representative blots from three independent experiments are shown; (**b**,**c**) Band intensities were quantified by ImageJ software and indicated as relative folds of iNOS/α-tubulin and COX-2/α-tubulin. Data are represented as the mean ± SD (*n* = 3). Statistical differences are presented ** *p* < 0.01 compared with the vehicle control (without LPS) and ## *p* < 0.01 compared with the LPS-treated vehicle.

Western blot analysis was employed to determine whether CR or ET exerted NO inhibition on activated microglia by blocking iNOS expression. Housekeeping protein α-tubulin was used as a loading control. Figure 2a,b shows that LPS (10 ng/mL) dramatically increased iNOS protein expression after 16 h treatment, by about 9.8-fold. Both CR and ET (0.05 and 0.1 mg/mL) attenuated LPS-mediated iNOS expression in dose-dependent manners.

Prostaglandin E_2_ (PGE_2_) is produced by activated microglia through the enzymatic action of cyclooxygenase (COX)-2 and prostaglandin E synthases (PGES) [38]. We thus investigated whether CR and ET could inhibit the LPS-induced protein expression of COX-2. Figure 2a,c shows that treatment of BV2 cells with LPS (10 ng/mL) for 16 h increased COX-2 protein expression by about 1.8-fold, as compared with the vehicle, and co-treatment with CR (0.05 and 0.1 mg/mL) and ET (0.1 mg/mL) markedly repressed LPS-mediated COX-2 up-regulation.

### 2.3. Effects of Ethanol Extracts of C. rutidosperma and E. thymifolia on mRNA Expression of iNOS and COX-2

**Figure 3:**
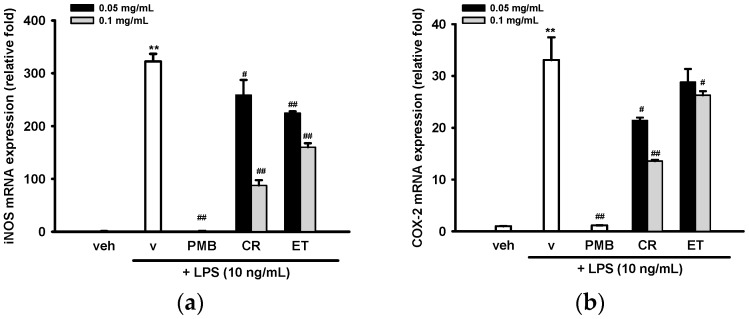
Effects of ethanol extracts of *C. rutidosperma* and *E. thymifolia* on the mRNA expression of iNOS and COX-2 in LPS-treated BV2 cells. BV2 cells were cultured with indicated reagent in six-well plates for 4 h followed by RNA extraction and RT-Q-PCR for measuring mRNA levels of iNOS (**a**) and COX-2 (**b**), as described in Materials and Methods. Data are represented as the mean ± SD (*n* = 3). Statistical differences are presented ** *p* < 0.01 compared with the vehicle control (without LPS) and # *p* < 0.05; ## *p* < 0.01 compared with the LPS-treated vehicle.

To investigate how CR and ET affect mRNA levels of iNOS and COX-2 in LPS-treated BV2 cells, RT-Q-PCR were employed. Transcriptome analysis shows that most of inflammatory genes were overexpressed 4 h after LPS stimulation in BV2 cells [33]. Similarly, we found that treatment of BV2 cells with LPS (10 ng/mL) for 4 h significantly upregulated the iNOS transcript level by 322-fold (Figure 3a). CR and ET dose-dependently inhibited iNOS mRNA expression. The iNOS mRNA levels correlated well with protein levels, indicating that CR and ET inhibited LPS-mediated nitric oxide production via repression of iNOS expression, predominantly at the transcription stage in BV2 cells.

LPS also strongly stimulated COX-2 mRNA expression after 4 h incubation in BV2 cells. In parallel to the results found for COX-2 protein expression, co-treatment of cells with LPS and CR (0.05 and 0.1 mg/mL) or ET (0.1 mg/mL) significantly attenuated COX-2 induction (Figure 3b). This result indicates down-regulation of COX-2 expression by CR and ET in BV2 cells occurs mainly at the transcription level.

### 2.4. Effects of Ethanol Extracts of C. rutidosperma and E. thymifolia on Pro-inflammatory Cytokine Production and Expression

**Figure 4:**
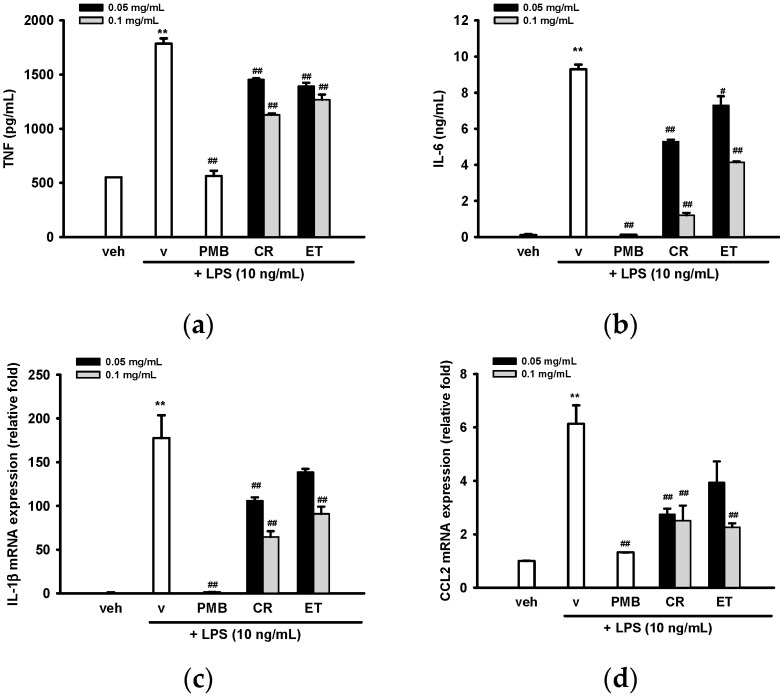
Effects of ethanol extracts of *C. rutidosperma* and *E. thymifolia* on the production and expression of pro-inflammatory cytokines in LPS-treated BV2 cells. (**a**,**b**) BV2 cells were treated with indicated reagent for 16 h and the supernatant were collected for TNF and IL-6 assay, as described in Materials and Methods; (**c**,**d**) BV2 cells were cultured with indicated reagent in six-well plates for 4 h followed by RNA extraction and RT-Q-PCR, as described in Materials and Methods. Data are represented as the mean ± SD (*n* = 3). Statistical differences are presented ** *p* < 0.01 compared with the vehicle control (without LPS) and # *p* < 0.05; ## *p* < 0.01 compared with the LPS-treated vehicle.

Tumor necrosis factor (formerly known as TNF-α), interleukin-6 (IL-6) and IL-1β are the major pro-inflammatory cytokines produced by LPS-activated macroglia [39]. We found that LPS induced TNF and IL-6 production in BV2 cells and co-treatment with CR and ET (0.05–0.1 mg/mL) could dose-dependently inhibit their production (Figure 4a,b). Figure 4c shows that LPS (10 ng/mL) treatment for 4 h caused a 177-fold increase in IL-1β mRNA expression. Co-treatment with CR and ET markedly attenuated LPS-mediated IL-1β over-expression. 

CCL2 (CC chemokine ligand 2)/MCP-1 (monocyte chemoattractant protein-1) is one of the most important chemokines for regulating the migration and infiltration of microglial cells [40]. A significant induction of CCL2/MCP-1 transcription was observed 4 h after LPS (10 ng/mL) treatment and co-treatment with CR and ET effectively inhibited its expression (Figure 4d).

### 2.5. Ethanol Extracts of C. rutidosperma and E. thymifolia Induce Nrf2 Target Gene Expression

**Figure 5:**
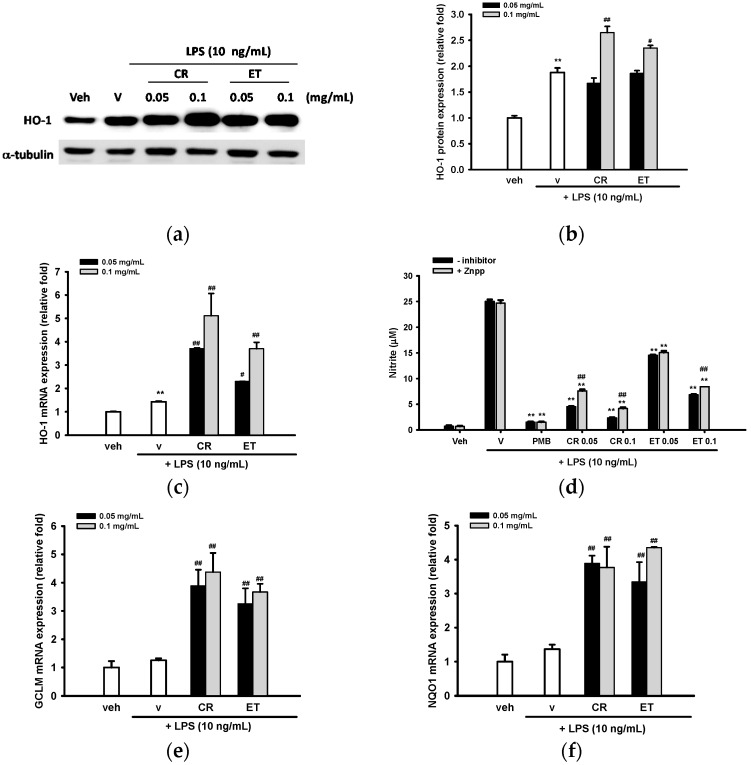
Induction of Nrf2 target gene expression by ethanol extracts of *C. rutidosperma* (CR) and *E. thymifolia* (ET). (**a**) BV2 cells were cultured with indicated reagent for 16 h and level of HO-1 expression was determined by Western blotting; (**b**) Band intensities were quantified by ImageJ software and indicated as relative fold of HO-1/α-tubulin; (**c**,**e**,**f**) HO-1, GCLM and NQO-1 mRNA expression levels were determined at 4 h after LPS treatment by RT-Q-PCR. Data are represented as the mean ± SD (*n* = 3). Statistical differences are presented ** *p* < 0.01 compared with the vehicle control (without LPS) and # *p* < 0.05; ## *p* < 0.01 compared with the LPS-treated vehicle; (**d**) BV2 cells were pre-treated with Znpp (5 μM) for 30 min, followed by PMB (10 μg/mL), CR or ET (0.05 and 0.1 mg/mL) treatment for further 30 min, prior to LPS (10 ng/mL) challenge for 20 h. NO production was determined by the Griess reagent. Data are represented as the mean ± SD (*n* = 3). Statistical differences are presented ** *p* < 0.01 compared with the LPS-treated vehicle and ## *p* < 0.01 compared with relative Znpp-untreated group.

Heme oxygenase 1 (HO-1), displaying established antioxidant and anti-inflammatory properties, is a known Nrf2 target gene [41]. Accumulating evidence indicates that HO-1 upregulation works against inflammatory responses, especially iNOS expression, in macrophages and microglia [9,11,42,43]. Figure 5a,b shows that LPS (10 ng/mL) slightly induced HO-1 protein expression by 1.9-fold, and co-treatment with 0.1 mg/mL CR and ET significantly enhanced HO-1 upregulation. However, a lower concentration of CR or ET (0.05 mg/mL) did not affect HO-1 expression.

Resembling HO-1 protein expression, LPS only slightly induced HO-1 mRNA expression after 4 h treatment, and co-treatment with CR and ET enhanced the induction dose-dependently (Figure 5c). Therefore, it is possible that CR and ET upregulated HO-1 expression primarily at the transcription level in BV2 cells.

To verify the role of HO-1 on the anti-NO production action of CR and ET, zinc protoporphyrin (ZnPP) IX, an HO-1 inhibitor, was added into BV2 cells for 30 min, followed by addition of CR and ET for a further 30 min, prior to LPS (10 ng/mL) insult for 20 h. Figure 5d shows that ZnPP (5 μM) did not affect NO production in vehicle control or LPS-treated cells. On the other hand, in the presence of LPS, combination of ZnPP and CR (0.05 and 0.1 mg/mL) or ET (0.1 mg/mL) significantly attenuated NO inhibitory action of CR and ET without affecting cell viability (Figure S1). These results indicate that HO-1 activity participated only in part in the anti-NO function of CR and ET.

We further investigated the mRNA expression of other Nrf2 target genes, the modifier subunit of glutamate cysteine ligase (GCLM) and NAD(P)H quinone dehydrogenase 1 (NQO1) [5]. Figure 5e,f shows that LPS alone did not induce GCLM or NQO1 transcription; however, co-treatment with CR and ET (0.05 and 0.1 mg/mL) markedly stimulated GCLM and NQO1 mRNA expression by three- to four-fold. This observation indicates that CR and ET activate Keap1-Nrf2 signaling pathway so as to adapt to oxidative stress.

### 2.6. Effects of Ethanol Extracts of C. rutidosperma and E. thymifolia on LPS-Mediated Activation of MAPK and NF-κB

**Figure 6:**
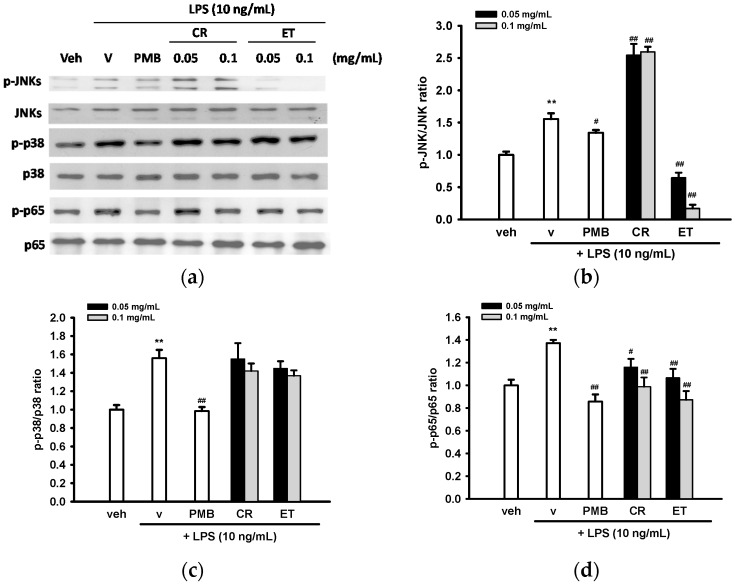
Effects of ethanol extracts of *C. rutidosperma* (CR) and *E. thymifolia* (ET) on phosphorylation of JNK, p38 MAPK and p65 in BV2 cells. (**a**) BV2 cells were pre-treated with the indicated reagent for 30 min and then stimulated for 30 min with LPS (10 ng/mL). Western blots were performed using the appropriate antibodies. Representative blots from three independent experiments are shown; (**b**–**d**) Densitometry is presented as the mean ± SD of three independent experiments. Statistical differences are presented ** *p* < 0.01 compared with the vehicle control (without LPS) and # *p* < 0.05; ## *p* < 0.01 compared with the LPS-treated vehicle.

The above described finding prompted us to investigate whether CR or ET treatment regulates MAPK or NF-κB activation, which is involved in the transcription of pro-inflammatory genes [44]. Treatment of BV2 cells with LPS (10 ng/mL) for 30 min stimulated JNK and p38 MAPK activation in BV2 cells (Figure 6a–c). CR significantly stimulated LPS-induced JNK phosphorylation but ET reduced its activation. Neither CR nor ET significantly changed LPS-mediated p38 (Figure 6a,c) or ERK activation in BV2 cells (data not shown).

To evaluate whether CR and ET affect NF-κB activation, BV2 cell lysates were probed for the antibody specific for Ser^536^ phosphorylation of the p65 NF-κB subunit. The results showed that LPS treatment for 30 min elevated p65 phosphorylation and co-treatment with PMB (10 µg/mL) attenuated its activation in BV2 cells. CR and ET could attenuate phosphorylation of p65 dose-dependently in BV2 cells (Figure 6a,d).

**Figure 7:**
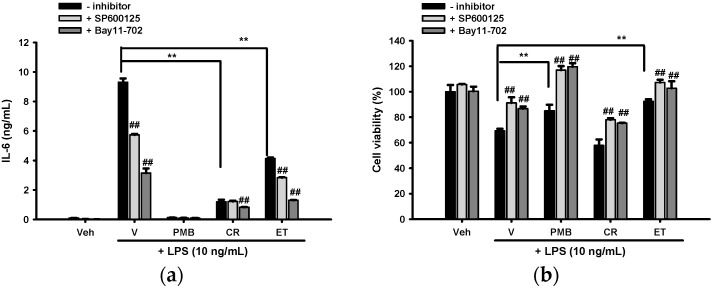
Effects of signaling inhibitors on the anti-IL-6 effects of CR and ET. (**a**) BV2 cells were pretreated for 30 min with inhibitor (SP600125, 10 μM; Bay 11-7082, 10 μM) followed by CR or ET (0.1 mg/mL) for 30 min before LPS (10 ng/mL) insult for 16 h. (**a**) Supernatant was recovered for IL-6 analysis; (**b**) Cell viability was analyzed by MTT assay. Data are represented as the mean ± SD (*n* = 3). Statistical differences are presented ** *p* < 0.01 compared with the LPS-treated vehicle and ## *p* < 0.01 compared with relative no inhibitor group.

To determine whether modulation of JNK or NF-κB activation relates to the anti-inflammatory activity of CR or ET, BV2 cells were pretreated with inhibitor of each pathway, SP600125, a JNK inhibitor, and Bay 11-7082, an inhibitor of IκB-α phosphorylation, for 30 min and then incubated with 0.1 mg/mL CR or ET for 30 min followed by LPS (10 ng/mL) for 16 h before analyzing IL-6 production and cell viability. Figure 7a shows that SP600125 and Bay 11-7082 (10 µM) significantly reduced LPS-mediated IL-6 production by about 39% and 67%, respectively, indicating that activation of the JNK and NF-κB pathways is involved in IL-6 production. Furthermore, the inhibitory effect of CR on IL-6 production was significantly enhanced by Bay 11-7082, but not by SP600125, as compared with no inhibitor control (*p* < 0.01). This result indicates that the attenuation of NF-κB activation by CR, but not the modulation of JNK signaling, participates in its anti-inflammatory action. In contrast, the inhibitory effect of ET on IL-6 production was augmented by the addition of both SP600125 and Bay 11-7082, indicating suppressed NF-κB activation and JNK phosphorylation many contribute to ET’s anti-inflammatory effects. Figure 7b shows that SP600125 and Bay 11-7082 (10 μM) alleviated LPS-mediated cytotoxicity in all test groups significantly (*p* < 0.01). This result indicates the involvements of JNK and NF-κB signaling in the LPS-mediated cytotoxicity in BV2 cells.

## 3. Discussion

Herbs are the main components of Traditional Chinese Medicine (TCM) and Traditional Indian Medicine (Ayurveda) and have long been used to treat various diseases. In recent years, increased popularity of complementary and alternative medicine has attracted growing scientific investigation in evaluating and understanding the pharmacological activities and mechanisms of herbal medicines. Previously, Bose et al. described that oral administration of CR resulted in significant analgesic, anti-inflammatory, and antipyretic activity in animal models [23]. Garipelli et al. showed that ET exhibited anti-inflammatory function using carrageenan-induced paw edema system [28]. In this study, we reported for the first time the molecular mechanism underlying the anti-neuroinflammatory effects of CR and ET. Our data indicate that they exert anti-inflammatory activities in BV2 microglia via down-regulation of pro-inflammatory gene expression, induction of antioxidant gene expression and modulation of activation of MAPK and NF-κB.

A growing body of evidence shows that microglia-mediated CNS inflammation plays an important role in the initial stages of neurodegenerative pathology [45]. BV2 cells have been extensively used as an alternative model system for primary microglia [33,36,46]. In this study, we found that CR and ET (0.05 and 0.1 mg/mL) significantly suppressed both iNOS expression and NO production dose-dependently in LPS-treated BV2 cells. The inhibition of NO production observed in this work may be attributed to the repression of iNOS mRNA and protein expression. CR and ET also caused significant attenuation in COX-2 mRNA and protein expression in LPS-treated BV2 cells. These observations indicate that CR and ET may inhibit PGE_2_ production in BV2 cells. Therefore, CR and ET have beneficial effects by attenuation of microglial activation and subsequent production of pro-inflammatory cytokines and mediators.

Pro-inflammatory cytokines, TNF, IL-6 and IL-1β, are the initiators of the inflammatory response. Overproduction of pro-inflammatory cytokines plays a key role in the neuroinflammation. This report demonstrates that CR and ET inhibited production of TNF and IL-6 in dose-dependent manners. CR and ET also decreased LPS-mediated mRNA expression of IL-1β and chemokine CCL2 dose-dependently. 

Nrf2 activation is crucial in modulating redox homeostasis and regulating inflammatory conditions via the induction of many stress responsive and cytoprotective enzymes including HO-1, GCLM and NQO-1 [47]. Here we found the ability of CR and ET to induce HO-1, GCLM and NQO-1 expression, indicating they may act as Nrf2 activators. Addition of Znpp, a competitive inhibitor of HO-1, partially attenuated the anti-NO effects of CR and ET, supporting the anti-inflammatory role of HO-1 [48]. From current results, we found that CR exhibited stronger inhibition than ET against production of pro-inflammatory cytokines and mediators in LPS-treated BV2 cells. This may be due to the fact that CR was more effective than ET in downregulation of pro-inflammatory gene expression and upregulation of anti-inflammatory HO-1 expression.

It is well-known that inflammatory stimuli could activate JNK, p38 MAPK and NF-κB [49]. The IκK-mediated Ser^536^ phosphorylation of p65 is important for activation of the canonical NF-κB pathway, and is also required for nuclear translocation of p65 and its acetylation in nucleus [50]. Many of the currently used anti-inflammatory drugs impair the function of NF-κB, thereby putting the screening of inhibitors of NF-κB activation, especially IκK inhibition, in the center of new drug development [51]. In this report, we found that the underlying signaling pathways for the anti-inflammatory activities of CR and ET are possibly through downregulation of p65 NF-κB phosphorylation in BV2 cells. Nrf2 and NF-κB redox signaling pathways control opposite cellular processes that lead to either cytoprotection or pathological development [52]. There are lots of anti-inflammatory or anti-carcinogenic phytochemicals that suppress NF-κB signaling and also activate the Nrf2 pathway. This phenomenon suggests a crosstalk between suppression of NF-κB signaling and activation of Nrf2 pathway may exist [53].

It has been reported that ET contains quercetrin, cymol, carvacrol, 2-sesquiterpenes, salicylic acid, steroids, terpenoids, glycosides, essential oils, minerals, tannins, and large number of phenolics [26]. However, as yet there are no reports regarding the chemical composition of CR. Therefore, the next step is isolation and identification of the lead compounds responsible for the anti-neuroinflammatory activities from CR and ET.

## 4. Materials and Methods

### 4.1. Materials

*C. rutidosperma* and *E. thymifolia* were purchased from a traditional Chinese herbal medicine store in Tainan, Taiwan in August 2015. The voucher specimens (CR001 and ET001) were deposited in the Department of Cosmetic Science, Chia Nan University of Pharmacy and Science (Tainan, Taiwan).

### 4.2. Extraction

The dried *C. rutidosperma* and *E. thymifolia* were ground into powder. The powder was immersed in a 95% ethanol solution for four times at room temperature. Then, the extract was filtered, evaporated on a rotary vacuum evaporator and finally lyophilized. The extracts of *C. rutidosperma* (yield: 12.8%) and *E. thymifolia* (yield: 7.1%) were kept at 4 °C. Before each experiment, the extracts were dissolved in 95% ethanol and sonicated for 2 min at room temperature.

### 4.3. BV2 Cell Culture and Measurement of Nitrite, TNF and IL-6 Release

Immortalized mouse microglial cell line BV2, which was developed in the laboratory of Professor E. Blasi [54], was kindly provided by Professor Shun-Fen Tzeng (Department of Biotechnology, National Cheng Kung University, Tainan, Taiwan). BV2 cells were cultured in Dulbecco’s Modified Eagle Medium/Nutrient Mixture F-12 (DMEM/F12, Invitrogen Life Technologies, Carlsbad, CA, USA) with 10% fetal bovine serum (HyClone, Logan, UT, USA), 100 U/mL penicillin, and 100 µg/mL streptomycin. Cells were maintained in a humidified incubator at 37 °C in 5% CO_2_.

BV2 cells (1 × 10^6^/mL) were pretreated with antibiotic polymyxin B (PMB, Sigma-Aldrich, St. Louis, MO, USA) or test reagent for 30 min prior to LPS (*Escherichia coli* O111:B4, Sigma-Aldrich) insult. TNF and IL-6 in the supernatant were measured by Mouse TNF and IL-6 ELISA Sets (BD Biosciences, San Diego, CA, USA) according to the manufacturer’s instructions. Nitrite (NO_2_^−^), which is one of two primary, stable and nonvolatile breakdown products of nitric oxide (NO), was determined using the Griess reagent (1% sulphanilamid and 0.1% naphthylenediamine in 5% phosphoric acid, Sigma-Aldrich) and A_550_ was measured against a sodium nitrite standard reference curve.

### 4.4. Cell Viability

Cell viability was assessed by MTT (3-(4,5-dimethylthiazol-2-yl)-2,5-diphenyltetrazolium bromide) tetrazolium reduction assay [55].

### 4.5. Western Blotting Analysis

RIPA buffer (Thermo Fisher Scientific, Inc., Rockford, IL, USA) was used to prepare cell lysates. The protein concentration was determined by the Bradford method (Bio-Rad Laboratories, Hercules, CA, USA) using bovine serum albumin as a standard reference.

Cell lysates were first separated on 8%–12% SDS-PAGE and then electroblotting onto polyvinylidene difluoride (PVDF) membranes (Hybond-P, GE Healthcare, Pittsburgh, PA, USA) using CAPS buffer system (10 mM CAPS pH 10.5, 10% (*v*:*v*) methanol) at 20 volts overnight at 4 °C. The membranes were blocked in freshly made blocking buffer (5% skim milk in PBS with 0.05% Tween 20, pH 7.4) for 6–8 h at room temperature. Blots were probed with specific primary antibody (Table 1) at a dilution of 1:1000–1:5000 overnight at 4 °C. Suitable horseradish peroxidase-conjugated secondary antibody (Jackson ImmunoResearch, West Grove, PA, USA) at a dilution of 1:10,000 was then added and incubated for 1 h before enhanced chemiluminescence detection (Amersham ECL Prime Western Blotting Detection Reagents, GE Healthcare). Some blots were stripped with Restore stripping solution (Thermo Fisher Scientific) before reprobed with another primary antibody. The intensity of the band was analyzed with ImageJ software (National Institutes of Health, Bethesda, MD, USA).

### 4.6. RNA Extraction and Reverse Transcription Real-Time PCR

Illustra RNA spin Mini RNA Isolation Kit (GE Healthcare) was used to isolate total RNA from BV2 cells. First strand cDNA was synthesized from 0.8 µg of isolated RNA using a High-Capacity cDNA Archive kit (Thermo Fisher Scientific). In real-time PCR step, cDNA was amplified using a Power SYBR Green PCR Master Mix (Thermo Fisher Scientific) with an appropriate primer pair (Table 2) in an ABI StepOne Real Time PCR System. PCR conditions were: 95 °C for 2 min, 40 cycles at 94 °C for 15 s, and 60 °C for 60 s. The relative mRNA expression were normalized with β-actin expression and then calculated by the 2^−ΔΔ*C*t^ method. The identity and purity of the amplified product was checked through analysis of the melting curve.

### 4.7. Statistical Analysis

Data were presented as means ± SD and analyzed by the Kruskal–Wallis Test. A *p* value of <0.05 was taken to be significant. If the Kruskal–Wallis Test showed a significant difference, then pairwise comparisons would be analyzed by the Mann–Whitney *U* Test.

## 5. Conclusions

The present study found that CR and ET treatment of BV2 microglial cells inhibited LPS-induced NO production by suppressing iNOS protein expression. CR and ET also caused attenuation in COX-2 mRNA and protein expression. CR and ET inhibited the production of pro-inflammatory cytokines, such as TNF and IL-6, and suppressed IL-1β and CCL2 transcriptional activity. The anti-inflammatory effects of CR and ET may be attributed, at least in part, to overexpression of antioxidant enzymes. The anti-inflammatory activities of CR and ET are possibly through downregulation of p65 NF-κB phosphorylation and/or modulation of JNK activation in BV2 cells.

## Figures and Tables

**Table 1:** Primary antibodies used in Western blotting.

Antibody	Company	Catalog Number
α-tubulin	Sigma-Aldrich	T6199
NOS	Cell Signaling (Danvers, MA, USA)	2977
COX-2	Santa Cruz (Santa Cruz, CA, USA)	Sc-166475
HO-1	Stressgen (San Diego, CA, USA)	SPA-895
Phospho-JNK1/2 (clone 81E11)	Cell Signaling	4668
JNK2 (clone 56G8)	Cell Signaling	9258
Phoshpo-p38 MAPK(clone 3D7)	Cell Signaling	9215
p38 MAPK	Cell Signaling	9212
Phospho-p65 NF-κB (clone 93H1)	Cell Signaling	3033
p65 NF-κB	Cell Signaling	3034

**Table 2:** Primer pairs used in RT-Q-PCR.

Gene	Primers (5′–3′)	Amplicon (bp)
*β-actin*	GGCTGTATTCCCCTCCATCG CCAGTTGGTAACAATGCCATGT	154
*iNOS*	GTTCTCAGCCCAACAATACAAGA GTGGACGGGTCGATGTCAC	127
*COX-2*	TGAGCAACTATTCCAAACCAGC GCACGTAGTCTTCGATCACTATC	74
*IL-1β*	TTCAGGCAGGCAGTATCACTC GAAGGTCCACGGGAAAGACAC	75
*CCL2*	TTAAAAACCTGGATCGGAACCAA GCATTAGCTTCAGATTTACGGGT	121
*HO-1*	AAGCCGAGAATGCTGAGTTCA GCCGTGTAGATATGGTACAAGGA	100
*GCLM*	AAGCCCAGGATTGGGTGCCG GGTCGGTGAGCTGTGGGTGT	141
*NQO1*	AGGATGGGAGGTACTCGAATC TGCTAGAGATGACTCGGAAGG	127

## Supplementary Materials

Supplementary materials can be found at www.mdpi.com/1422-0067/17/9/1420/s1.
