# Supplementary material for: Cleome rutidosperma and Euphorbia thymifolia Suppress Inflammatory Response via Upregulation of Phase II Enzymes and Modulation of NF-κB and JNK Activation in LPS-Stimulated BV2 Microglia
Source: Int J Mol Sci. 2016 Aug 27;17(9):1420. doi: 10.3390/ijms17091420 (PMC5037699; doi:10.3390/ijms17091420)
Supplement: Supplementary file 1 [file ijms-17-01420-s001.pdf]

Supplementary Materials: *Cleome rutidosperma* and *Euphorbia thymifolia* Suppress Inflammatory Response via Upregulation of Phase II Enzymes and Modulation of NF-κB and JNK Activation in LPS-Stimulated BV2 Microglia

Hsiou-Yu Ding Pei-Shan Wu and Ming-Jiuan Wu

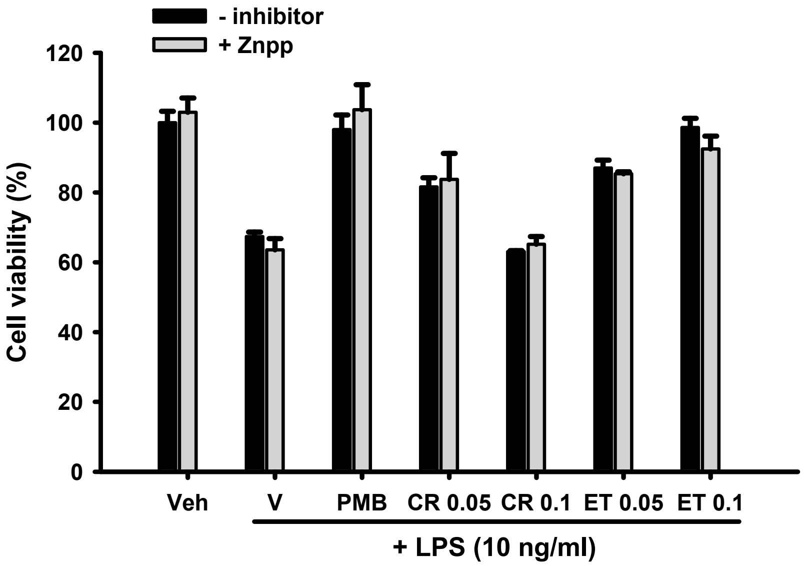

Figure S1. Effect of HO-1 inhibitor, Znpp, on the cell viability of BV2. BV2 cells were pre-treated with Znpp (5 μM) for 30 min, followed by PMB (10 μg/mL), CR or ET (0.05 and 0.1 mg/mL) treatment for further 30 min, prior to LPS (10 ng/mL) challenge for 20 h. Cell viability was determined by MTT assay. Data are represented as the mean ± SD (n = 3).
